# Efavirenz- but not nevirapine-based antiretroviral therapy decreases exposure to the levonorgestrel released from a sub-dermal contraceptive implant

**DOI:** 10.7448/IAS.17.4.19484

**Published:** 2014-11-02

**Authors:** Kimberly Scarsi, Mohammed Lamorde, Kristin Darin, Sujan Dilly Penchala, Laura Else, Shadia Nakalema, Pauline Byakika-Kibwika, Saye Khoo, Susan Cohn, Concepta Merry, David Back

**Affiliations:** 1 Department of Pharmacy Practice, University of Nebraska Medical Center, Omaha, NE, USA; 2 Infectious Diseases Institute, Makerere University College of Health Sciences, Kampala, Uganda; 3 Division of Infectious Diseases, Feinberg School of Medicine, Northwestern University, Chicago, IL, USA; 4 Department of Molecular and Clinical Pharmacology, University of Liverpool, Liverpool, UK; 5 Department of Pharmacology and Therapeutics, Trinity College Dublin, Dublin, Ireland

## Abstract

**Introduction:**

Sub-dermal hormone implants, such as levonorgestrel (LNG), are a safe and desirable form of long-acting contraception, but their use among HIV-positive women on antiretroviral therapy (ART) may be compromised given the potential for a cytochrome P450 3A-mediated drug–drug interaction. Our study aimed to characterize the pharmacokinetics of LNG released from a sub-dermal implant over six months in HIV-positive Ugandan women on nevirapine (NVP)- or efavirenz (EFV)-based ART.

**Material and Methods:**

This non-randomized, parallel group study compared LNG pharmacokinetics between HIV-positive Ugandan women not yet eligible for ART (control group, *n*=18) and those on stable NVP- (*n*=20) or EFV- (*n*=20) based ART. The two-rod (75 mg/rod) LNG sub-dermal implant was inserted at study enrolment. LNG sampling was obtained pre-implant and at weeks 1, 4, 12 and 24 post-insertion. LNG concentrations were analyzed using a validated LC-MS/MS method, with an assay calibration range of 50–1500 pg/mL. Safety monitoring, including a pregnancy test, was conducted at each study visit.

**Results:**

At enrolment, participants had a mean age of 31 years; CD4+ cell counts were similar between the control, NVP and EFV groups (758, 645 and 568 cells/mm^3^, respectively; *p*=0.09); all women in the NVP and EFV groups had an undetectable HIV-RNA. Women in the control group had a higher baseline body weight (73 kg) compared to those in the NVP (63 kg; *p*=0.03) or EFV groups (60 kg; p<0.01). By linear regression, weight was a significant predictor of LNG concentrations (1 kg increase in weight=5 pg/mL decrease in LNG, *p*=0.03). LNG concentrations are reported in the table.

**Conclusions:**

Over a 24-week period, LNG concentrations were 40–54% lower in women on EFV-based ART, despite their having a significantly lower body weight, compared to those not on ART. In women on NVP-based ART, LNG concentrations were 32–39% higher than those observed in the control group, a difference partially explained by body weight. These data confirm a significant drug interaction occurs between the LNG implant and EFV, adding to growing concern for reduced contraceptive efficacy with their combined use. In contrast, these data support use of the LNG implant with NVP-based ART.

**Table 1:** LNG concentrations shown as geometric mean (GM) with 90% confidence intervals (CIs)

	Control group; pg/mL	NVP group; pg/mL	NVP:control GM ratio (90% CI)	EFV group; pg/mL	EFV:control GM ratio (90% CI)
Week 1	1003 (720, 1286)	1326 (1073, 1579)	1.32 (1.22, 1.49)	462 (370, 553)	0.46 (0.43, 0.51)
Week 4	629 (496, 761)	866 (737, 995)	1.38 (1.31, 1.48)	349 (268, 429)	0.55 (0.54, 0.56)
Week 12	547 (433, 661)	758 (656, 860)	1.39 (1.30, 1.52)	326 (268, 386)	0.60 (0.58, 0.62)
Week 24	500 (394, 605)	679 (569, 790)	1.36 (1.30, 1.44)	280 (208, 353)	0.56 (0.53, 0.58)

During this 24-week period, no participant discontinued the LNG implant due to adverse events and no pregnancy events occurred.

